# A Synergistic Perspective on Multivariate Computation and Causality in Complex Systems

**DOI:** 10.3390/e26100883

**Published:** 2024-10-21

**Authors:** Thomas F. Varley

**Affiliations:** Vermont Complex Systems Center, University of Vermont, Burlington, VT 05405, USA; tfvarley@uvm.edu

**Keywords:** Berkson’s paradox, synergy, multivariate information theory, higher-order interactions, partial information decomposition

## Abstract

What does it mean for a complex system to “compute” or perform “computations”? Intuitively, we can understand complex “computation” as occurring when a system’s state is a function of multiple inputs (potentially including its own past state). Here, we discuss how computational processes in complex systems can be generally studied using the concept of statistical synergy, which is information about an output that can *only* be learned when the joint state of all inputs is known. Building on prior work, we show that this approach naturally leads to a link between multivariate information theory and topics in causal inference, specifically, the phenomenon of causal colliders. We begin by showing how Berkson’s paradox implies a higher-order, synergistic interaction between multidimensional inputs and outputs. We then discuss how causal structure learning can refine and orient analyses of synergies in empirical data, and when empirical synergies meaningfully reflect computation versus when they may be spurious. We end by proposing that this conceptual link between synergy, causal colliders, and computation can serve as a foundation on which to build a mathematically rich general theory of computation in complex systems.

## 1. Introduction

What does it mean to say that a complex system, such as the brain, “computes”? The metaphor of computation is a canonical one throughout the field of complex systems [1,2] although unlike digital and analog computers designed by engineers, complex systems are self-organizing, noisy, and often continuous. While early cyberneticians made use of concepts like Turing machines and Boolean logic gates as analogies [3], as the field has progressed, it has become increasingly clear that complex systems are not merely elaborate Turing machines. Formal theories that rely on mathematical notions of well-defined, computable functions are often too rigid for the noisy, multifaceted, ill-defined, and non-deterministic world of complex systems. As a result, like many other topics in the field of complex systems, applying the concept of computation often relies on the intuition of researchers rather than objective quantification.

Despite this, it is also clear that many systems *do* seem to do something very much like the most general theories of computation. Consider a neuron: it is well known that neurons receive inputs from pre-synaptic parent neurons (in the form of excitatory and inhibitory neurotransmitters) and account for those inputs when “deciding” (computing) their behavior in the immediate future (whether to fire an action potential or not). Here, we suggest a relaxed and pragmatic definition of computation, in the spirit of the simple mapping account [4,5], that incorporates these basic features. In the context of complex systems, we operationalize computation as occurring when a system, or subset of a system, accounts for the state of multiple exogenous or endogenous inputs when deciding its next state. While this definition is very general, this may ultimately be a virtue; it does not require that the process of accounting for inputs be mathematically well defined, nor does it assume a discrete, finite state machine, and it works just as well for continuous systems as discrete ones. For instance, an economist might say that a business computes the optimal price of its products as some nebulous function of inputs (costs of material, competitors’ pricing, etc).

The specific mechanisms and physical implementations of these computations are all different, but they share a common high-level feature: information about the states of many elements, possibly including the past state of the computer itself, is integrated to “decide” on a given next state, out of some space of possibilities. We might call this the “integrated information” account of computation (note that “integrated information” in this context is not necessarily the same as integrated information theory, which is a proposed theory of consciousness).

Here, we show that this definition of computation is particularly useful in the context of multivariate information theory [6,7,8], which concerns itself with the interactions between three or more variables (higher-order interactions). For such an account to be useful, particularly one as general as this, it would be useful if it pointed to some analytic tools that scientists could use to try and understand the computational structure of whatever system they are interested in. Based on the integrated information account of computation, we would expect that an observer attempting to model a putative computational process would find that predicting the output requires knowing the joint state of all of the inputs simultaneously. This notion is formalized by the concept of *synergy*: information about a target that is only disclosed by the joint state of all of the inputs and no simpler combination of sources [9].

Much of this work is inspired by the developments of Lizier et al. [10], who proposed that “non-trivial” computation could be recognized by multivariate dependencies that were “greater than the sum of their parts”. This approach was further refined by Lizier, Flecker, and Williams in 2013 [11], who combined their method with the recently proposed approach using the partial information decomposition (explained in Section 2.2.1) as a principled measure of synergy. Subsequently, Timme et al. proposed using synergy as a measure of computation in neocortical circuits [12], an approach that has led to a series of studies in the area of computational neuroscience [13,14,15,16]. While highly productive, many instances of this approach have relied on intuitive arguments relating synergy to computation, and the formal connections have been largely unexplored. In this paper, we propose to refine the mathematical links between synergy and the integrated information account of computation, showing that the two can be linked to another set of fundamental concepts in complex systems science: collider bias and causal inference.

## 2. Berkson’s Paradox, Colliders, and “Computation”

Berkson’s paradox is one of the most well-known veridical paradoxes in statistics and is often used to demonstrate the effects of non-random sampling of data [17]. The usual presentation is as follows:

Alice is a social psychologist who is interested in how desirable traits (intelligence, attractiveness, etc.) are distributed over the U.S. population. Her data include information from a large number of Hollywood actors (a convenience sample; talent scores can be inferred from movie reviews and attractiveness from tabloids). Using movie reviews and tabloids, every actor is assigned a score for both talent and attractiveness. When Alice plots acting talent against attractiveness, she finds a statistically significant negative correlation. With a *p*-value ≤0.05 in hand, Alice rushes off to publish a paper about how attractiveness and acting talent are anticorrelated and then writes a grant proposing a research program into how the genes that code for acting talent and facial attractiveness are antagonistic.

**Figure 1:**
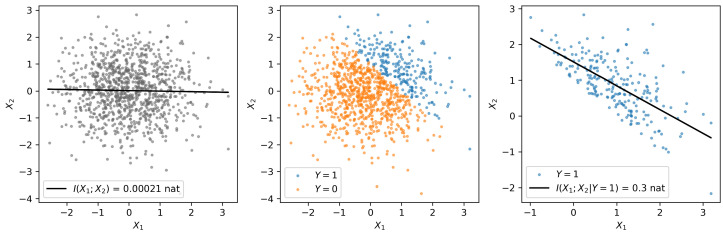
**Left:** Two random, normally distributed variables $X_{1}$ and $X_{2}$ with zero mean, unit variance, and $X_{1}⊥X_{2}$. There is no mutual information between them. In the example of movie stars, $X_{1}$ and $X_{2}$ might be analogous to attractiveness and talent. **Center:** we can define a third random variable as a threshold function: $Y=(X_{1}+X_{2})>1$, analogous to the “selection” criteria that define who will be a movie star as a function of whether they reach some minimal threshold of attractiveness and/or talent. **Right:** If we then compute the mutual information between $X_{1}$ and $X_{2}$ conditional on Y=1, we see a spurious correlation, suggesting that $X_{1}$ and $X_{2}$ are anticorrelated, when they are actually independent. This is Berkson’s paradox.

When phrased in this somewhat cartoonish way, Alice’s mistake is fairly obvious: she has an extremely biased sample (indeed, Berkson’s paradox is sometimes called selection bias [18]). Hollywood celebrity is fickle; however, it is generally true that to reach the status of celebrity, one must be either unusually attractive or unusually talented. Ugly and untalented people do not (typically) make it to the silver screen, and so Alice’s data are fatally compromised from the outset. Thinking in terms of computation, we might say that “the media system” is “computing” whether a person reaches some threshold baseline of joint talent and attractiveness to be allowed onto the red carpet (and, by extension, onto Alice’s list). For a geometric visualization of Berkson’s paradox, see Figure 1. In the next section, we will discuss the usual interpretation of Berkson’s paradox in the context of collider bias and causal inference, before moving on to an alternative interpretation that relates collider bias and Berkson’s paradox to higher-order information sharing and synergy.

### 2.1. The Usual Interpretation: Collider Bias

Berkson’s paradox appears to be about two variables (such as attractiveness and acting talent) but is, in fact, actually about a higher-order interaction between three variables (one of which is “hidden”). This third variable is whether a given member of the population is selected to be in Alice’s dataset or not. Importantly, the state of this hidden variable, *Y*, is itself a function of the two other variables we are interested in. Both talent and attractiveness causally influence an actor’s success. In the case of Alice’s study, the inclusion criteria could be a threshold function that computes whether someone is talented enough or attractive enough to make it to Hollywood stardom.

Written out in the language of causal diagrams (directed, acyclic graphs, or DAGs), we can represent our system of three variables as a *collider*:

(1) $$X_{1}\to Y\leftarrow X_{2}$$

Colliders are something of a bugbear in modern causal inference and multivariate statistics [19]. We will discuss the problem of causal structure inference in more detail below, but briefly, when attempting to understand the structure of a system, the presence of colliders makes it difficult to understand when there is real dependence between two variables, or if they are actually (“causally”) independent and the empirical dependence is illusory. Much has been written on the problem of collider bias, but from an intuitive perspective, the source of the bias is fairly simple. When we sample data from a system with a collider, we are unwittingly creating an unbiased sample. If we then try and learn correlations between features, we are actually computing the conditional mutual information $I(X_{1};X_{2}|Y)$ when we might believe that we are computing the bivariate mutual information $I(X_{1};X_{2})$. The subtlety emerges when we realize that conditioning the mutual information is not a purely subtractive operation (which it is commonly believed to be). It is entirely possible for the conditional mutual information to be greater than the unconditioned mutual information. In the language of causal diagrams, Pearl might say that conditioning on the collider opens a path between $X_{1}$ and $X_{2}$ [20]: an illusion of dependency has been created, one that may not be there in the original dataset.

### 2.2. The Synergistic Interpretation

In the example of Alice and the movie stars, the two input variables (talent and attractiveness) are a priori independent (i.e., $I(X_{1};X_{2})=0$ bit), but when conditioned on the third variable (whether they are attractive enough, or talented enough, to make it in Hollywood), a significant dependency appears (i.e., $I(X_{1};X_{2}|Y)>>0$ bit). A very natural question to ask might be how different is the true dependency between $X_{1}$ and $X_{2}$ from the conditional dependency? We can compute the simple difference as follows: $I\left(X_{1};X_{2}\right)-I\left(X_{1};X_{2}|Y\right)$.

For readers already familiar with information theory, this relationship should be recognizable. It defines the *co-information* [21], one of three proposed multivariate generalizations of the classic, bivariate, Shannon mutual information:

(2) $$\begin{array}{cc} Co(X_{1};X_{2};Y) & =I\left(X_{1};X_{2}\right)-I\left(X_{1};X_{2}|Y\right)\\ \; & =I\left(X_{1};Y\right)-I\left(X_{1};Y|X_{2}\right)\\ \; & =I\left(X_{2};Y\right)-I\left(X_{2};Y|X_{1}\right) \end{array}$$

As a generalized mutual information, co-information is interesting because it does not obey the non-negativity of either the bivariate mutual information or the two other generalizations (both the total correlation [22] and the dual total correlation [23] are strictly non-negative). Let us first imagine the case where $Co(X_{1};X_{2};Y)>0$ bit. If the co-information is positive, then $I\left(X_{1};X_{2}\right)>I\left(X_{1};X_{2}|Y\right)$. In this case, learning the state of *Y* “explains away” the dependency between $X_{1}$ and $X_{2}$. There must be some uncertainty that is “triply redundant” between all three variables, such that learning the state of any individual variable accounts for correlations between the other two (consider the symmetry of the three equations in Equation (2)). The case of the negative co-information is a little bit harder. Clearly, this occurs when $I\left(X_{1};X_{2}\right)<I\left(X_{1};X_{2}|Y\right)$, which implies that learning *Y* somehow “illuminates” (or perhaps “hallucinates”) a dependency between $X_{1}$ and $X_{2}$ that is not visible when the two are considered on their own. Interpreting this phenomenon (which is widespread in empirical data [24]) has been a standing puzzle in information theory since co-information was introduced in the 1950s. Various information theorists have proposed a variety of interpretations and decompositions, including in terms of joint and conditional mutual information, sums and differences of entropies [25], or lattice structures [26]. However, it was not until the development of the partial information decomposition (PID) by Williams and Beer in 2010 that the negativity of the co-information was finally interpreted in terms of purely higher-order interactions [9].

The PID (explained below) reveals that the co-information is negative (and by extension, Berkson’s paradox occurs) when there is information about *Y* that is only learnable when the state of the rest of the system (i.e., $X_{1}$ and $X_{2}$) is known jointly. We will argue below that, in the context of a collider where *Y* is causally influenced by both $X_{1}$ and $X_{2}$, this is indicative of a “computational process” happening in *Y*, where multiple streams of information are integrated as part of *Y*’s “choice” of what state to adopt.

#### 2.2.1. Explaining Partial Information Decomposition

The partial information decomposition (PID) is a framework for assessing how the interaction of many “input” variables reveals information about the state of a target variable [9,27]. As previously mentioned, the PID was not the first attempt to study different orders of information sharing in the case of multiple sources and a single target (see [25] for an early example), but the PID is unique in that it revealed the entire structure of multivariate information and formalized the notion of multiple “kinds” of higher-order interaction. For two inputs and the target, it is straightforward to compute the joint mutual information that both sources provide about the target as follows:

(3) $$I\left(X_{1},X_{2};Y\right)=H\left(Y\right)-H\left(Y|X_{1},X_{2}\right)$$

However, this scalar value does not reveal the structure of the information: What information about *Y* could only be learned by observing $X_{1}$? Or only by observing $X_{2}$? What information could be learned by observing either $X_{1}$ alone or $X_{2}$ alone? What “synergistic” information can only be learned by observing $X_{1}$ and $X_{2}$ jointly (and no simpler combination of elements)? The PID was proposed in a seminal paper by Williams and Beer [9] and decomposes the joint mutual information into a set of non-overlapping “partial information atoms”:

(4) $$\begin{array}{cc} I(X_{1},X_{2};Y)= & Red\left(X_{1},X_{2};Y\right)+Syn\left(X_{1},X_{2};Y\right)+ \end{array}$$

(5) $$\begin{array}{cc} & \;Unq\left(X_{1};Y/X_{2}\right)+Unq\left(X_{2};Y/X_{1}\right) \end{array}$$

In the two-variable case, these four terms describe every possible dependency between inputs and the target. The redundant information $Red(X_{1},X_{2};Y)$ is the information that is duplicated over $X_{1}$ and $X_{2}$ such that it could be learned by observing either $X_{1}$ alone or $X_{2}$ alone. The unique information term $Unq(X_{i};Y/X_{j})$ is the information about *Y* that could only be learned by observing $X_{i}$. Finally, the synergistic information $Syn(X_{1},X_{2};Y)$ is the information about *Y* that can only be learned when the states of $X_{1}$ and $X_{2}$ are observed together and, crucially, cannot be learned by any simpler set of sources.

The marginals’ mutual information can be similarly decomposed as follows:

(6) $$\begin{array}{c} I\left(X_{1};Y\right)=Red\left(X_{1},X_{2};Y\right)+Unq\left(X_{1};Y/X_{2}\right) \end{array}$$

(7) $$\begin{array}{c} I\left(X_{2};Y\right)=Red\left(X_{1},X_{2};Y\right)+Unq\left(X_{2};Y/X_{1}\right) \end{array}$$

**Figure 2:**
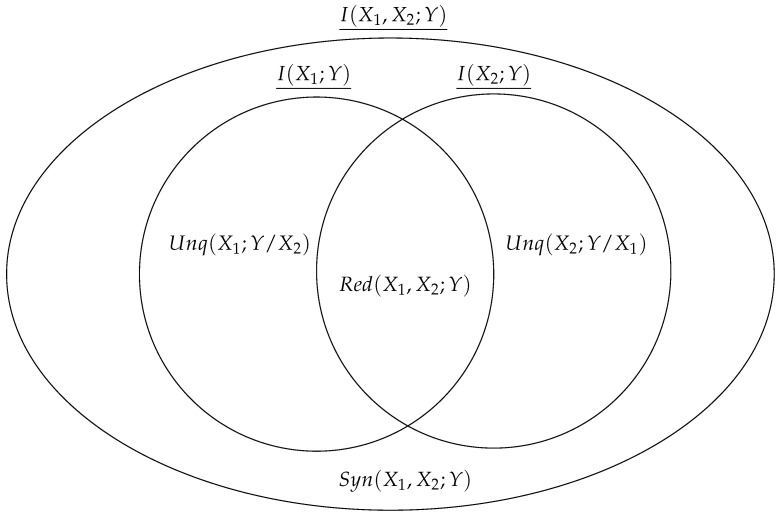
Visual intuition behind the bivariate PID.

Note here that context is important: since we are analyzing the bivariate relationship $X_{i}-Y$ in the context of the collider $\{X_{1},X_{2}\}\to Y$, the redundant information $Red(X_{1},X_{2};Y)$ still counts toward $I(X_{i};Y)$. For a visualization of the PID using Venn diagrams, see Figure 2.

There remains an active debate about the best way to define redundancy. Unlike the Shannon entropy, which is uniquely specified by Shannon’s original axioms, many different possible redundancy functions can be shown to satisfy the initial Williams and Beer axioms. These functions can return meaningfully different results (for further discussion, see [28,29]); thus, in any given analysis of empirical data, care should be taken when choosing one or another. By and large, however, the arguments made in this paper do not require operationalizing a specific measure. In cases where we want to compute a PID for didactic purposes (see Section 2.3), we use the minimum mutual information [30]:

(8) $$Red\left(X_{1},X_{2};Y\right):=\min_{i}I\left(X_{i};Y\right)$$

Intuitively, we can understand the minimum mutual information and estimate how much uncertainty, on average, about *Y* would be resolved regardless of whether we learn $X_{1}$ alone or $X_{2}$ alone. Once again, this is not the only viable measure and has a number of limitations; however, it is also conceptually the simplest, which suits our purposes here. One significant benefit of this redundancy function is that it is known to be analytically correct for multivariate Gaussian variables [31] and has the nice property that it only depends on the pairwise, marginal relationship between $X_{i}$ and *Y* (for more, see [32,33]).

#### 2.2.2. Decomposing Conditional Mutual Information and Co-Information

With the PID established, it is possible to gain new insights into measures like co-information and conditional mutual information. For example, consider the following identity:

(9) $$I\left(X_{1};X_{2}|Y\right)=I\left(X_{1};X_{2},Y\right)-I\left(X_{1};Y\right)$$

If we substitute in Equations (4) and (6), we find

(10) $$\begin{array}{cc} I(X_{1};X_{2}|Y) & =Unq\left(X_{1};X_{2}/Y\right)+Syn\left(X_{1},Y;X_{2}\right) \end{array}$$

(11) $$\begin{array}{cc} \; & \;=Unq\left(X_{1};X_{2}/Y\right)+Syn\left(X_{2},Y;X_{1}\right) \end{array}$$

where $Unq(X_{1};X_{2}/Y)$ refers to the unique information about $X_{2}$ disclosed by $X_{1}$ *in the context of Y*.

We can see that the effect of conditioning on the collider is to make visible the higher-order interaction between all three variables: $Syn(X_{1},Y;X_{2})$ is the synergistic information about $X_{2}$ that is only learnable when $X_{1}$ and *Y* are known together (and likewise for $Syn(X_{2},Y;X_{1})$). This provides the crux of why conditioning on a collider can cause dependencies to appear: the observer suddenly gains access to higher-order information (in the form of the synergistic dependency) that they can only “see” when only considering $X_{1}$, $X_{2}$, and *Y* together.

We can further refine this intuition by considering the following co-information:

(12) $$Co\left(X_{1};X_{2};Y\right)=I\left(X_{1};X_{2}\right)-I\left(X_{1};X_{2}|Y\right)$$

Decomposing the co-information with respect to the target, *Y* reveals that

(13) $$Co\left(X_{1};X_{2};Y\right)=Red\left(X_{1},X_{2};Y\right)-Syn\left(X_{1},X_{2};Y\right)$$

We can see that, whenever Berkson’s paradox occurs and $Co(X_{1};X_{2};Y)<0$, synergy outweighs redundancy. Said differently, information about the state of *Y* is dominated by an “integrated” interaction between multiple sources. *Y* is “choosing” its state as a function of multiple inputs. Accordingly, we say that synergy is reflecting this high-order computation. This link between Berkson’s paradox and synergy was first noted by Rosas et al. [34] in an excellent study of higher-order information sharing. Here, we elaborate on this crucial insight by extending it using the PID, as well as discussing how it may be applied in empirical contexts.

### 2.3. Case Study I: Summing Dice

To demonstrate the proposed link between synergy and computation, it helps to go through a simple example. Here, we have (arguably) the simplest “computation” possible: addition. To model it, consider two fair, six-sided dice, $D_{1}$ and $D_{2}$, with $D_{1}⊥D_{2}$. We will create a third variable *S*, such that $S=D_{1}+D_{2}$. We hope it is not controversial to say that *S* implements a computation (in this case, addition). While the distributions of $D_{1}$ and $D_{2}$ are maximally entropic, this is not the case for *S*: the probability that S=2 is 1/36 (since there is only one combination of inputs that leads to that outcome). In contrast, the probability that S=6 is 1/6 since there are six possible combinations of inputs that sum to 6.

By construction, we know that $I(D_{1};D_{2})=0$ bit. However, $I(D_{1};D_{2}|S)\approx 1.896$ bit, and so $Co(X_{1};X_{2};Y)\approx -1.896$ bit. The synergistic component of the information dominates and so the co-information is negative and Berkson’s paradox has occurred!

If we compute the actual PID using the minimum mutual information function for redundancy, we find that $Red(D_{1},D_{2};S)\approx 0.689$ bit and $Syn(D_{1},D_{2};Y)\approx 2.585$ bit (and both unique terms are 0 bit exactly since the dice are identical). Where do these numbers come from? Intuitively, we can understand the non-zero redundancy by noticing that, even though $D_{1}⊥D_{2}$, learning the state of either die alone is enough to “rule out” some possible states of *S*. For example, learning that $D_{1}=1$ or $D_{2}=1$ is sufficient to exclude all possible values of S>7, since to achieve such a value requires that at least one die be greater than or equal to two. This is what Harder refers to as “mechanistic redundancy”, in contrast to “source redundancy”, which is attributable to correlations between the inputs [35].

The remaining information about *S* is synergistic. This is because completely resolving all the uncertainty about the state of *S* requires knowing both inputs. Learning that $D_{1}=1$ is sufficient to rule out any S>7; however, it is not enough to specify the value of *S* any further. Doing so requires knowing both inputs, and this is the nature of computation. We should note that we are not saying that synergistic information *is* computation in any fundamental sense. Whatever physical process that reads the value of the individual dice and computes the sum is what is performing the computation. The synergistic information is merely a kind of statistical fingerprint, indicating that information has been integrated in some higher-order fashion.

### 2.4. Case Study II: Transfer Entropy

The perspective that synergistic information represents some putative notion of “computation” can help us make sense of difficult-to-interpret phenomena in complex systems research. As a second case study, let us consider the transfer entropy [36,37]. The transfer entropy from some variable *X* to another variable *Y* is defined as follows:

(14) $$TE\left(X\to Y\right)=I\left(X_{past};Y_{t}|Y_{past}\right)$$

where $X_{past}$ refers to the (potentially multivariate) embedded past of *X*, $Y_{past}$ refers to the (potentially multivariate) embedded past of *Y*, and $Y_{t}$ refers to the state of *Y* at time *t*. The logic behind the transfer entropy is to capture that information about *Y*’s next state disclosed by *X*’s past that is above and beyond the information disclosed by *Y*’s own past (hence the conditioning).

As we have already established, conditioning mutual information is not a purely subtractive manipulation. Generally, the tacit assumption implicit in applications of the transfer entropy is that conditioning on $Y_{past}$ will reduce the apparent correlation between $X_{past}$ and $Y_{t}$. However, this is not always the case: how do we interpret the case of $I\left(X_{past};Y_{t}\right)<I\left(X_{past};Y_{t}|Y_{past}\right)$?

Williams and Beer [38] showed that the transfer entropy can be decomposed into

(15) $$TE\left(X\to Y\right)=Unq\left(X_{past};Y_{t}/Y_{past}\right)+Syn\left(X_{past},Y_{past};Y_{t}\right)$$

The first term, $Unq(X_{past};Y_{t}/Y_{past}),$ is generally understood to refer to the true bivariate information transfer and was termed the “state-independent transfer entropy” (SITE) by Williams and Beer, as it does not depend on the past state of the target. The second term, $Syn(X_{past},Y_{past};Y_{t}),$ is less clear: it refers to the information about $Y_{t}$ that can only be learned when both *X*’s past and *Y*’s past are known together. Williams and Beer termed it the “state-dependent information transfer” (SDTE) since it does depend on the past state of the target.

This decomposition shows that the putatively bivariate transfer entropy actually conflates pairwise and higher-order dependencies (this is also generally true of any conditional mutual information). This discovery prompted intense criticism of the transfer entropy as a technique for complex network inference by James and Crutchfield [39], as it obviously challenges the interpretation that TE(X→Y) measures unidirectional information “flow”. Consequently, they argue that the transfer entropy cannot be properly localized to a single source. Since the critique, however, this facet of the bivariate transfer entropy has received little attention, despite its significance. Recently, Daube et al. [40] showed that the SDTE contributes to the estimation of transfer entropy from simulated data. Furthermore, contra Barrett [31], who suggested stripping the SDTE and using only the “true” bivariate SITE transfer, Daube et al. argued that the SITE actually does worse than the SDTE.

Based on both empirical findings and theoretical arguments presented here, it seems implausible to continue to treat the transfer entropy as a measure of conveniently bivariate information flow. However, none of the existing treatments meditate on how to best intuitively understand what kinds of dynamics are represented by the SDTE. Formally, the synergy is well understood and easy to derive. However, a unifying interpretative framework has largely been lacking. If we take that synergy refers to integrative computation, then the SDTE starts to become a little bit less mysterious: any process that “decides” its next state as a function of some combinations of memory and external perturbation can be modeled as performing a “computation” on two inputs: one is the state of its own past, and one is the value of the input it receives. State-dependent transfer entropy could be thought of as reporting that part of the transfer entropy reflects the computation that the target element performs in deciding its next state as a function of what is stored in its memory and what input it receives from another part of the system.

## 3. Computation and Causality in Complex Systems

So far, we have assumed that the causal model of the collider is known, and notions such as “inputs” and “outputs” are well specified. In the case of summing the values of the dice, the inputs ($D_{i}$s) are obviously the individual dice, and the output (or target, *S*) is the sum of the input. In this context, it is obvious that the relevant information decomposition is of $I(D_{1},D_{2};S)$, and the notion of synergy makes intuitive sense. Now suppose that we have three new variables, $A_{1}$, $A_{2}$ and *B*, and that rather than being related by a collider motif, they are related by a broadcasting motif as follows:

(16) $$A_{1}\leftarrow B\to A_{2}$$

where *B* broadcasts its state to each $A_{i}$. In this case, Berkson’s paradox will not hold: if $A_{1}$ and $A_{2}$ are otherwise independent, when conditioning on *B*, we would expect $I(A_{1};A_{2}|B)=0$ bit. Given the definition of the co-information given in Equation (12), it is easy to see that $Co\left(A_{1};A_{2};B\right)=I\left(A_{1};A_{2}\right)$. Since $Co(A_{1};A_{2};B)>>0$ bit, we can infer that the information structure of the triad is, overall, dominated by redundancy. This is consistent with the intuition that a broadcaster is duplicating information over both $A_{1}$ and $A_{2}$ (as well as representing it in itself).

There is, however, an apparent problem. From Equation (13), we know that

(17) $$Co\left(A_{1};A_{2};B\right)=Red\left(A_{1},A_{2};B\right)-Syn\left(A_{1},A_{2};B\right)$$

If $Co(A_{1};A_{2};B)>>0$, then how might we interpret the possibility of non-zero synergy in this context? From a mathematical perspective, it is straightforward: there is uncertainty about the state of *B* that can only be resolved when $A_{1}$ and $A_{2}$ are both known. Unfortunately, this seems to complicate our argument that synergy is a statistical fingerprint of computation; the broadcaster motif does not seem to be performing any meaningful “computation”. It is merely duplicating information over downstream elements.

To resolve this uncertainty, it is important to remember that the PID is an inherently directed analysis, dividing the system into inputs and targets. As previously mentioned in the case of the collider, the inputs and targets map neatly onto the causal model of the collider itself. In the case of the broadcaster motif, this is not the case. While it is numerically possible to compute $Syn(A_{1},A_{2};B)$, doing so is running “against the causal flow” and, consequently, appears to be meaningless from a directed perspective. It could be said that the map and territory are in conflict: the orientation of the PID is misaligned with the causal structure of the system. This is important to highlight, as it shows that not all computable synergies are necessarily meaningful: the structure of the underlying causal model is a key consideration, and researchers interested in applying these analyses should be mindful of the different contextual factors that might change the interpretation of the numerical value.

What about in cases where the causal ground truth is not known? It is easy to construct tractable toy models in theoretical contexts, but in natural sciences, rarely is a blueprint handed to us by God. Instead, it must be inferred. Suppose we have some triad, {X,Y,Z}, which could be either a collider or a broadcaster and we do not know which. Furthermore, we lack any domain expertise that can be leveraged to assume a structure. In the material we have covered so far, disambiguating these two cases is difficult. We could look for Berkson’s paradox, assuming that if X→Z←Y then Co(X;Y;Z)<0, but even if the structure is a collider, if *X* and *Y* are correlated, we may still find that redundancy outweighs synergy. In systems that have a large synchronous component to their dynamics (for example, the nervous system), this is a real concern.

If a scientist has a dataset from which synergies can be computed, how could they disambiguate between those synergies that represent some kind of computational process (as in the collider) and those that do not (as in the broadcaster)? We argue that, ultimately, this is a question of causal inference [20] and leveraging both domain expertise, as well as more refined techniques for structure learning in complex systems [41,42] must be incorporated into the analysis. If, given some dataset, it is possible to construct a causal (or merely effective) scaffold of the system, then colliders can be identified as putative sites of computation. This approach has been leveraged in computational neuroscience: given finely resolved, temporally ordered spiking data, it is possible to infer effective connectivity networks using measures like the bivariate or multivariate transfer entropy [15,16], from which colliders can be sampled.

Alternately, if one has strong priors on the structure of the system under study, then those may be leveraged. For example, in a quantitative study of intersectionality in social systems, Varley and Kaminski studied how demographic factors such as race and sex synergistically disclose information about outcomes such as incomes [43]. In this context, there is ample prior evidence to assume a collider-like structure, while a broadcaster-like structure can be ruled out because there is no plausible way that income could causally impact features like race or sex.

Recently, considerable work has involved the problem of inferring directed models that account for higher-order interactions. It has long been known that strictly pairwise approaches to structure learning fail in the presence of higher-order interactions [8], and finding optimal ways to account for higher-order redundancies and synergies remains an open problem. One popular approach is the multivariate transfer entropy [44,45], which takes a serial conditioning approach to account for higher-order information in transfer entropy, although other approaches include the framework proposed by Mijatovic et al., which introduced a balance index (B-index) that quantified whether the interaction between two elements was redundancy-dominated or synergy-dominated in the context of the rest of the system. Beyond the pairwise network approach, representing higher-order information with hypergraphs has been recently explored [46,47] as well. Similarly, generalizations of the partial information decomposition to stochastic processes [48] can reveal time-directed higher-order information in dynamical systems.

Assuming that one is able to construct a plausible causal (or effective) model of the system under study, there is still the issue that the joint mutual information itself is not a causal measure. Previous work has established that even time-directed measures of information flow, such as temporal mutual information or transfer entropy, poorly reflect causal dependencies [49,50]. The link between information flow, causality, and structure inference is a rich field that we cannot completely survey (for further discussion, see [51,52,53]), and the problem of true causal information decomposition remains an outstanding issue. Ultimately, the question of attributing higher-order, synergistic causation may not have a unique solution but, like the question of redundancy, may depend on the specific way one operationalizes the notion of “causality” (interventional, counterfactual, etc). In the short term, however, we propose that a small modification of the joint mutual information may be useful here. In 2003, Tononi and Sporns proposed a measure they termed “effective information”, which is the mutual information from a source to a target when the entropy of the sources is maximal [54]:

(18) $$EI\left(X\to Y\right)=I\left(X^{H_{\max}};Y\right)$$

By forcing a maximum entropy distribution on the inputs, the effective information attempts to simulate random assignment in interventional approaches to causality. Later, Hoel et al. [52] argued that the effective information could be understood as an instance of Pearl’s do-calculus applied to mutual information: EI(X→Y)=I(Y;do(X)). Being modified mutual information, effective information can be decomposed using the PID, lending a more causal flavor to the synergy than is usually the case (as well as removing any possible source redundancy [35]). For an example of this approach to discrete data, see [43]. We should be very clear, however, that while effective information and do-calculus approximate a causal inference (and by extension, causal computation), they are still ultimately based on correlational analyses, which can break down in certain contexts. It is known that the Shannon entropy can misrepresent structures in complex systems [55]. For researchers interested in applying these methods, considering the appropriateness of a an information-theoretic model should be seen as a necessary first step in experimental design. Future work considering other notions of information structure (such as algorithmic information theory and algorithmic information dynamics [56]) will complement this work.

## 4. Discussion

Since the notion of synergy was formalized in the context of the PID, it has been widely applied to a number of different fields. Each of these applications brings with it distinct interpretations, and there is not much cross-talk between disciplines as to the general meaning of synergistic information. For example, when considering sociological identities, synergy is equated with the sociological concept of “intersectionality” [43]. In the context of biological neural networks, synergy has been associated with information processing in the nervous system [12,13,15,16,57,58], while in artificial neural networks, synergy has been associated with the complexity of representations [59] or multi-task learning [60]. Synergy has been identified in the cardiovascular system as well [48]. When considering econometric data, synergy has been associated with economic sophistication [61], and synergy has been found to correlate with the level of consciousness in patients who have been anesthetized or are comatose [58,62]. In climatological data, synergy has been identified, although its significance remains unclear [57,63,64], and in theoretical biology, synergy has been associated with notions of “individuality” or organismic autonomy [65].

This list is non-exhaustive, but it is sufficient to indicate that synergy is ubiquitous in the natural world; far from being some kind of strange or exotic dependency that only occurs in constructed models, synergy has been found in complex systems at almost every scale. Despite this ubiquity, the variety of different, context-dependent interpretations shows that science in general lacks a unified framework for understanding the significance of synergy generally. Here, we propose that synergy can be understood as a kind of fingerprint of computation in complex systems: when an element of a system “chooses” its next state based on some multi-argument function that accounts for multiple inputs, synergistic information will almost certainly exist. By showing that synergistic dynamics are intimately related to the notion of colliders in causal inference, we hope to ground future studies of synergistic dynamics in complex systems in a common framework.

Since the groundbreaking work of Williams and Beer first formalized the notion of synergy in the context of the PID over a decade ago [9], the majority of the focus of the field has been on solving the problem of how to recognize it in data. This has included work on a plethora of redundancy functions (for review, see [28,29]), extensions of the PID (such as the multi-target integrated information decomposition [66,67] and the partial entropy decomposition [68,69]), and the generalized information decomposition [70], as well as non-PID-based heuristics such as the O-information [71], the O-information rate O-information rate [48] (a derivative of the O-information specifically designed for dynamical system), or the α-synergy decomposition [72].

At this point, the problem of recognizing synergistic dependencies in data is, if not totally solved, well studied enough that the field has a rich toolkit of measures to apply to any given dataset. This puts us in the position of being able to move beyond asking merely “is there synergy?” or “where is the synergy?” Instead, we must begin asking “what is synergy for?”

### Conclusions

In this paper, we explored the idea that modeling “computation” in complex systems is related to the information-theoretic notion of statistical synergy. Synergy occurs when there is information about the state of an output variable that can only be learned when the joint state of all of its input variables is known and proposed that this is a statistical “fingerprint” reflecting some physical process by which an output element “chooses” its next state as a function of multiple inputs (rather than merely propagating information forward). To further formalize this notion, we related this process to the collider motif studied in the field of causal inference, where a single variable is causally influenced by multiple inputs simultaneously. We showed that the well-known collider bias (also known as Berkson’s paradox) occurs because of a synergistic interaction between the inputs and output, providing a mathematical link between the world of multivariate information theory and causal inference or structure learning. We further proposed that this link deepens our understanding of both statistical synergy as a concept and the phenomenon of computation in complex systems.

## Data Availability

No new data were created for this article.
